# Risk Factors for Myocardial Injury During Cardio‐Pulmonary Bypass‐Assisted Heart Surgery: A Retrospective Single‐Center Study

**DOI:** 10.1111/aas.70330

**Published:** 2026-09-23

**Authors:** Julie T. Holm, Sebastian C. Wiberg, Lars Grønlykke, Sarah E. Busch, Peter Hasse Møller‐Sørensen, Christian Hassager, Theis S. Itenov

**Affiliations:** ^1^ Department of Cardiothoracic Anesthesiology and Intensive Care Copenhagen University Hospital – Rigshospitalet Copenhagen Denmark; ^2^ Department of Anesthesia and Intensive Care Copenhagen University Hospital Bispebjerg and Frederiksberg Copenhagen Denmark; ^3^ Department of Clinical Medicine Faculty of Health and Medical Sciences, University of Copenhagen Copenhagen Denmark; ^4^ Department of Cardiology Copenhagen University Hospital – Rigshospitalet Copenhagen Denmark

## Abstract

**Introduction:**

Myocardial injury is a significant contributor to 30‐day mortality after cardiac surgery with cardiopulmonary bypass. We aimed to investigate the association between mean arterial pressure, norepinephrine use during the aorta cross‐clamp period, and aorta cross‐clamp duration with myocardial injury during cardiopulmonary bypass‐assisted open‐heart surgery.

**Method:**

We identified all adults (≥ 18 years) undergoing coronary artery bypass grafting on cardiopulmonary bypass at Rigshospitalet, Copenhagen, between January 1st 2018 and December 31st 2019. The primary outcome was the change from baseline in creatine kinase myocardial band during the first 18 postoperative hours. Exposures included mean arterial pressure, norepinephrine use during the aorta cross‐clamp period, and aorta cross‐clamp duration. Associations were investigated using linear mixed‐effect models.

**Results:**

A total of 1443 patients were included in the analysis. Patients treated with norepinephrine during the aortic cross‐clamp period had 19% (95% CI: 9.0%–29%; *p* < 0.001) higher creatine kinase myocardial band postoperatively compared with those not treated with norepinephrine. Higher mean arterial pressure was not significantly associated with creatine kinase myocardial band (3.0%, 95% CI: −2.0% to 9%; *p* = 0.20). Patients treated with norepinephrine with a mean arterial pressure above the median (39 mmHg) had similar creatine kinase myocardial band levels compared to patients not treated with norepinephrine with a mean arterial pressure below the median, with a difference of 2% (95% CI: −0.18% to 0.22%, *p* = 0.80). Creatine kinase myocardial band increased by 12% (95% CI: 10%–14%; *p* < 0.001) for each 15‐min increase in aortic cross‐clamp duration.

**Conclusion:**

This retrospective observational study suggested a possible link between norepinephrine use and myocardial injury during cardiopulmonary bypass‐assisted open‐heart surgery, while mean arterial pressure appeared to have no impact. Further randomized controlled trials are necessary.

**Editorial Comment:**

In this single center retrospective cohort analysis, intraoperative cardiac surgery management factors were analyzed along with postoperative cardiac injury marker levels. Associations for aortic cross‐clamp time, noradrenaline use during this, and mean arterial pressures are presented for post‐op creatine kinase MB levels.

## 1 Introduction

Myocardial injury following cardiopulmonary bypass (CPB) is a significant cause of death within the first 30 days after surgery [1, 2, 3]. Key procedural steps, such as connecting and disconnecting the patient from the CPB pump, cause circulatory changes, which can increase the risk of myocardial injury [4]. The duration of CPB is another well‐documented contributor to myocardial injury, and existing studies emphasize the risk of reperfusion injury after the removal of the aortic cross clamp (AoX) [5]. Hemodynamic management during CPB has evolved over time, particularly regarding mean arterial pressure (MAP) targets and the use of vasopressors/inotropes. In recent years, norepinephrine (NE) has, by expert consensus, become the first‐choice vasopressor for open‐heart surgery with CPB. However, the complex relationship between NE use and organ perfusion raises concerns [6, 7, 8, 9]. Furthermore, previous studies show conflicting results regarding MAP targets, which leaves us without a clear answer about the optimal MAP target during CPB‐assisted open‐heart surgery [8, 9, 10, 11, 12, 13, 14, 15].

Nevertheless, several perioperative factors, including surgical factors and circulatory management, are suspected to increase the risk of myocardial injury. However, the specific risk of individual contributing factors during CPB‐assisted open‐heart surgery remains to be investigated.

In this study, we aim to investigate the associations and interactions between myocardial injury and AoX duration, MAP, and the use of NE during the AoX period of CPB‐assisted open‐heart surgery. We hypothesized that an increase in AoX duration, a decrease in MAP during the AoX period, and the use of NE during the AoX period were associated with myocardial injury.

## 2 Material and Method

### 2.1 Study Design

This is an observational retrospective single‐center study of prospectively collected data from a tertiary heart center. We conducted and reported the study according to the STROBE statement [10].

We established a database containing all consecutive adult patients undergoing coronary artery bypass grafting (CABG) +/− valve surgery at Rigshospitalet from January 1st 2018 to December 31st 2019. Practice has not changed substantially since the data were collected. Data includes pre‐, intra‐, and postoperative information.

### 2.2 Patients

**FIGURE 1:**
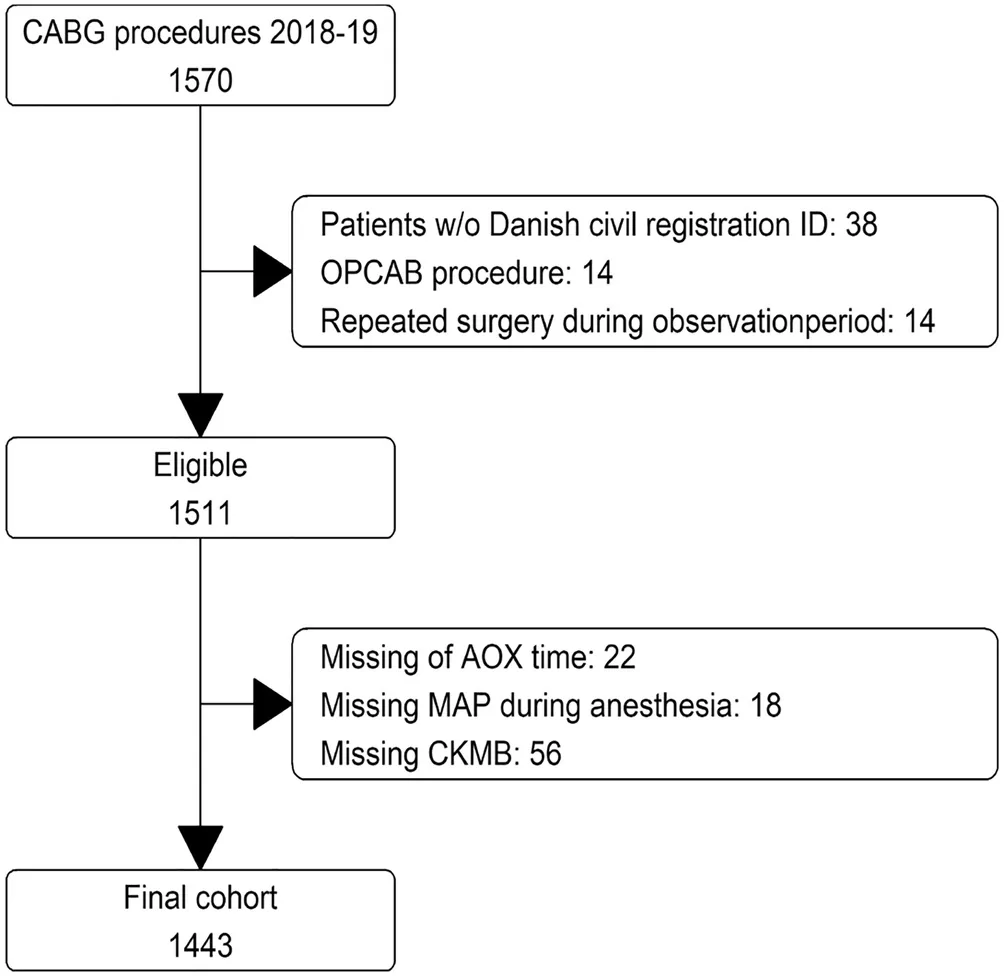
Flow diagram of patient inclusion, patients ≥ 18 years of age who underwent CABG surgery in the designated period. AoX, aortic cross‐clamp; CABG, coronary artery bypass‐grafting; MAP, mean arterial pressure; CKMB, creatine kinase—myocardial band; OPCAB, off‐pump coronary artery bypass. Definitions: Patients w/o Danish civil registration ID: Patients without a Danish civil registration number. AoX time: From aortic cross‐clamping to the removal of the aortic cross‐clamp.

All adults (age ≥ 18 years) were considered eligible for inclusion if they underwent CABG surgery in the designated period. The sample size was pragmatically determined by the number of consecutive patients undergoing CPB‐assisted open‐heart surgery during the study period, totaling 1570 procedures. We included both patients exclusively undergoing CABG surgery and combined procedures (CABG + concomitant procedures such as valve surgery). If a patient had multiple CABG surgeries, we included only the first operation. Patients without a Danish personal identification number were excluded. We excluded patients who underwent off‐pump coronary artery bypass procedures (OPCAB) (Figure 1).

### 2.3 Outcome

The primary outcome was the change in creatine kinase myocardial band (CK‐MB) during the first 18 postoperative hours. All patients had routine CK‐MB measurements taken at three standardized time points, which reduces the risk of confounding by indication. CK‐MB was analyzed as repeated measurements, allowing the intra‐patient trajectory of CK‐MB to be included in the analysis.

### 2.4 Exposures

AoX duration, MAP, and NE‐use were selected a priori as potential risk factors for myocardial injury during CPB‐assisted open‐heart surgery. AoX duration and MAP were analyzed as both above and below the median, and as continuous variables. We model exposure to represent change by increases of 15 min in AoX duration and 10 mmHg in MAP, which corresponds to changes in CK‐MB levels. The administration of NE was treated as a categorical variable (yes/no) in the primary analyses. To provide a more detailed clinical context, NE requirements were also reported as a continuous variable. We chose to primarily analyze MAP and NE in a defined period, from aortic cross‐clamping to the removal of the aortic cross‐clamp, referred to as the AoX‐period. In the Supporting Information, there will also be analyses of MAP and NE in the periods before and after the AoX‐period.

### 2.5 Anesthesia

Anesthesia was induced with fentanyl (20 μg/kg), propofol (0.5–2 mg/kg), and rocuronium (0.6–1.0 mg/kg). Maintenance of anesthesia was achieved with sevoflurane (0.5%–3%) and a continuous infusion of remifentanil (15–25 μg/kg/h).

MAP was monitored via a radial artery cannula, and the MAP goal was at the treating anesthesiologist's discretion. NE was administered, as deemed necessary by the attending anesthesiologist, to maintain stable hemodynamics.

### 2.6 Surgery

All procedures were undertaken through median sternotomy. After heparinization (350 IU/kg, ACT > 480 s), normothermic CPB (36.5°C–37.0°C bladder temperature) primed with Ringer‐acetate was initiated as follows: in the ascending aorta, an arterial cannula was placed, and a two‐stage venous cannula was inserted through the right atrial appendage. An antegrade cardioplegia cannula was placed near the aortic root.

The cardioplegia solution consisted of 84 mmol/L potassium, given at a 1:4 (potassium: blood) ratio, resulting in a potassium infusion of 16.8 mmol/L. The cardioplegia solution was cooled to 5°C using a heat exchanger (CSC14, Liva Nova) and a heater‐cooler unit (Stoerkert 3T, Liva Nova). The initial cardioplegia dose was 1000 mL, and thereafter a 350 mL dose was replenished every 20 min.

## 3 Statistical Analysis

Continuous variables were reported as the mean with standard deviation or the median with interquartile range, depending on the distribution. Categorical variables were reported as frequencies and percentages.

AoX duration and MAP were reported as medians. NE‐use was reported as categorical (yes/no) and grouped into three dose groups: NE doses 1 (0 to 0.01 μg/kg/min), 2 (0.01 to 1 μg/kg/min), and 3 (> 1 μg/kg/min).

We investigated the relationship between risk factors and CK‐MB levels (logarithmically transformed to approximate a normal distribution) using both univariate and multivariable linear mixed‐effects models. Patient ID was included as random effects. The primary outcome variable was the logarithm of CK‐MB (log CK‐MB), and the main exposure (fixed‐effects) variables were AoX duration, MAP, and NE. The multivariable analysis was adjusted for age, sex, and pre‐operative left ventricular ejection fraction. We investigated the interaction between NE and MAP through a two‐way interaction between NE administration (yes/no) and MAP above/below the median. The interaction test was done only in univariate analysis to maintain adequate statistical power and avoid overfitting. We had very few missing values, and we did complete case analysis.

Differences were considered statistically significant at *p* < 0.05. All analyses were performed in R Statistical Software version 4.2.2 (R Core Team, 2022).

## 4 Results

During the observation period, there were 1570 CABG procedures, and 1443 patients were included in the analysis. Reasons for exclusion are presented in Figure 1. Of the included patients, 217 (15%) had concomitant valve surgery. Baseline patient characteristics are presented in Table 1.

**TABLE 1:** Baseline characteristics of the study population of patients ≥ 18 years of age who underwent CABG surgery in the designated period.

Characteristics	*N*	Patients (%) (*n* = 1443)
Sex, *n* (%)	1443	
Female		220 (15)
Male		1223 (85)
Age (years), Median (Q1−Q3)	1443	67 (59 to 73)
Weight (kg), Median (Q1−Q3)	1442	84 (74 to 94)
Pre‐operative LVEF (%), Median (Q1−Q3)	1442	55 (45 to 60)
Number of coronary arteries involved, *n* (%)	1406	
LM		393 (28)
One vessel		97 (6.9)
Two vessel		241 (17)
Three vessel		675 (48)
LM stenosis, *n* (%)	1407	393 (28)
RCA stenosis, *n* (%)	1407	1125 (80)
CX stenosis, *n* (%)	1407	1116 (79)
LAD stenosis, *n* (%)	1407	1275 (91)
CABG and valve surgery, *n* (%)	1443	217 (15)
Aortic valve, *n* (%)	1443	172 (12)
Mitral valve, *n* (%)	1443	51 (3.5)
Tricuspid valve, *n* (%)	1443	9 (0.6)
Radialis graft, *n* (%)	1443	199 (14)
Smoking status, *n* (%)	1350	
Current		330 (24)
Former		661 (49)
Never		359 (27)
Diabetes type 1, *n* (%)	1443	9 (0.6)
Diabetes type 2, *n* (%)	1443	99 (6.9)
Hypertension, *n* (%)	1443	158 (11)
Chronic kidney disease, *n* (%)	1443	12 (0.8)
Creatinine (mmol/L), median (Q1−Q3)	1428	85 (74 to 99)
eGFR (CKD‐EPI, ml/min/1.72 m^2^), median (Q1−Q3)	1428	79 (65 to 90)
Albumin (g/L), median (Q1−Q3)	1035	37.0 (34.0 to 40.0)
Hemoglobin (mmol/L), median (Q1−Q3)	1429	8.70 (8.00 to 9.30)
Carbamide (mmol/L), median (Q1−Q3)	1383	5.70 (4.70 to 7.30)
C‐Reactive protein (mg/L), median (Q1−Q3)	1263	3 (1 to 7)
MAP, median = 40, *n* (%)	1443	
> 40 mmHg		584 (40)
≤ 40 mmHg		859 (60)

Abbreviations: CX, circumflex coronary artery; eGFR, estimated glomerular filtration rate; IQR, interquartile range; LAD, left anterior descending coronary artery; LM, left main coronary artery; LVEF, left ventricular ejection fraction; RCA, right coronary artery.

### 4.1 MAP and NE During the AoX‐Period

The postoperative CK‐MB concentration was 19% (95% CI: 9.0% to 29%; *p* < 0.001) higher in patients treated with NE in the AoX‐period compared to patients not treated with NE.

**FIGURE 2:**
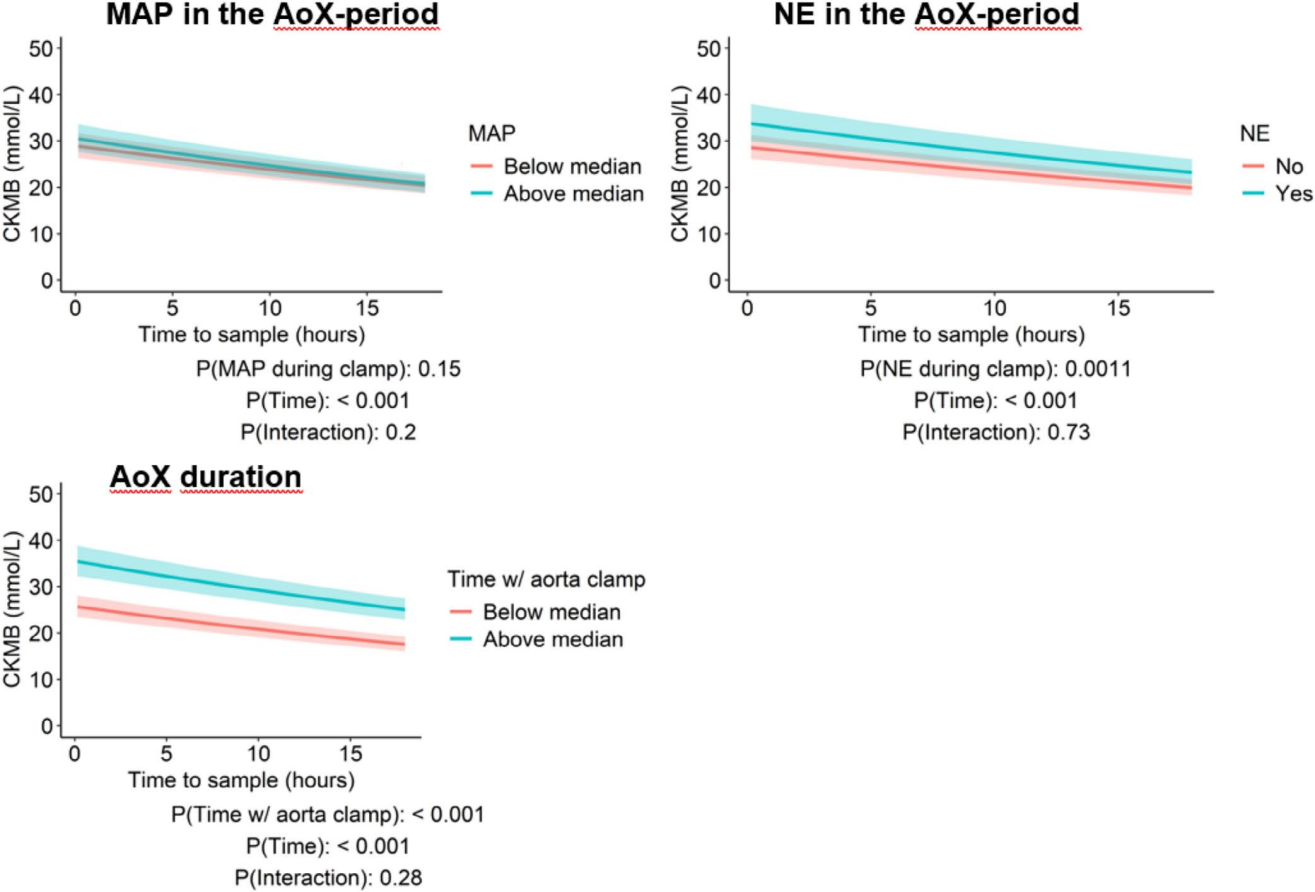
Risk factors for myocardial injury following cardiopulmonary bypass. Mean arterial pressure and norepinephrine in the AoX period, and AoX duration as risk factors for myocardial injury, indicated by the CK‐MB response within the first 18 h after surgery. CKMB, creatine kinase—myocardial band; MAP, mean arterial pressure; NE, norepinephrine. Model structure: All three risk factors are treated as dichotomous exposures. MAP and AoX duration are categorized as below and above the median, and NE is categorical yes/no. CK‐MB values were log‐transformed before analysis; thus, estimates represent the relative change in CK‐MB levels. Variables: MAP median (cut‐off) = 39 mmHg; Time W/aorta clamp median (cut‐off) = 49 min. Definition: During clamp: refers to the AoX‐period, which is defined as the duration from aortic cross‐clamping to the removal of the aortic cross‐clamp; Time W/aorta clamp: defined as the duration from aortic cross‐clamping to the removal of the aortic cross‐clamp; Time to sample (h): Time from the end of surgery to CK‐MB sample, which is the first 18 h postoperative.

In contrast, MAP during the AoX‐period, measured as a continuous variable, was not associated with postoperative CK‐MB levels (95% CI: −2% to 10%; *p* = 0.20). When separated into MAP above/below a median of 39 mmHg, there was no difference in CK‐MB levels postoperatively (Figure 2), (Table S1).

Analyzing NE as a continuous variable to understand its association with administered doses, patients receiving an NE dose of 0.01–1 μg/kg/min during the AoX‐period had an 18% higher CK‐MB level postoperatively than those who did not receive any NE during the same period (95% CI: 0.08% to 0.29%, *p* < 0.001). A stronger association was observed in patients receiving a dose above 1 μg/kg/min (95% CI: 0.08% to 0.38%, *p* < 0.001) (Table S6, Figure S1), who exhibited a 23% higher CK‐MB level postoperatively compared to patients who did not receive NE during the AoX‐period.

### 4.2 MAP and NE Interaction Test

**FIGURE 3:**
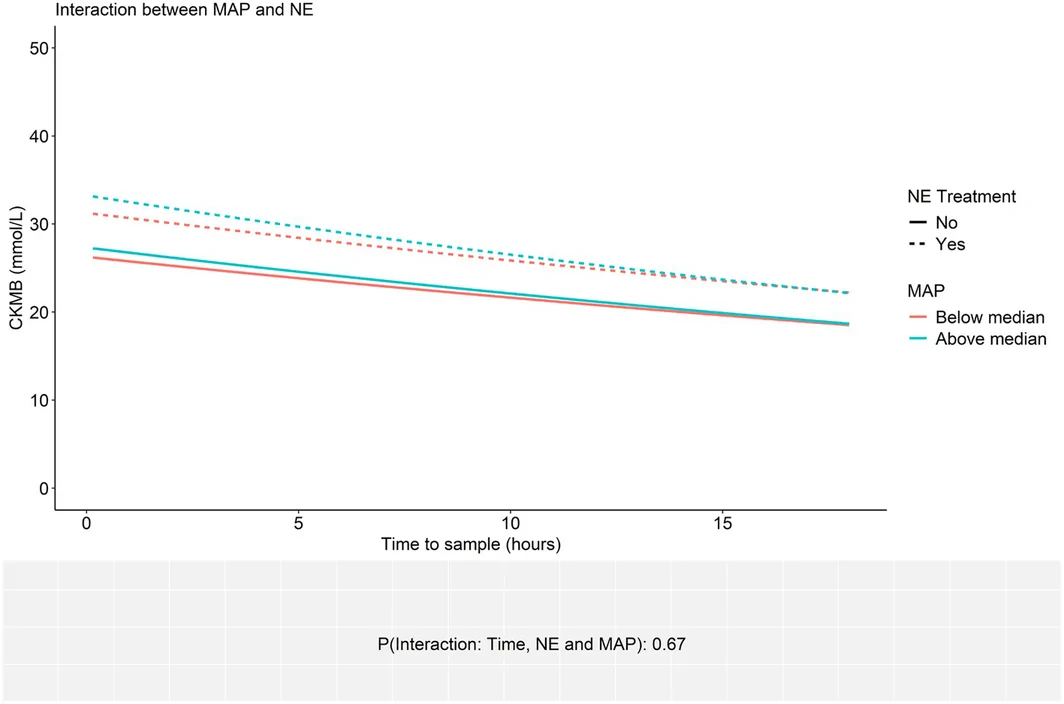
Interaction between norepinephrine and mean arterial pressure in the AoX‐period. MAP and NE during the AoX‐period and the association with the CK‐MB response during the first 18‐h postoperative—an interaction analysis between NE and MAP. MAP: Mean Arterial Pressure; NE: Norepinephrine; CKMB: Creatine Kinase—Myocardial Band; MAP Median (Cut‐Off) = 39 mmHg. Model structure: Data are presented below and above the median for MAP. CK‐MB values were log‐transformed before analysis; thus, estimates represent the relative change in CK‐MB levels. Variables: MAP median (cut‐off) = 39 mmHg. Definitions: Time to sample (h): Time from the end of surgery to CK‐MB sample, which is the first 18 h postoperative. AoX‐period: From aortic cross‐clamping to the removal of the aortic cross‐clamp.

When dividing the population by the median MAP of 39 mmHg (35 to 43 mmHg), we found that patients who received NE and had a MAP above the median had similar CK‐MB levels to those who did not receive NE and had a MAP below the median. The difference in CK‐MB levels between these groups was 2.0% (95% CI: −18% to 22%; *p* = 0.80) (Figure 3), (Table S5).

### 4.3 AoX Duration

The median AoX duration was 49 min (IQR: 36 to 64 min) (Table 2).

**TABLE 2:** Characteristics of surgical risk factors and mean arterial pressure during the AoX‐period of patients ≥ 18 years of age who underwent CABG surgery in the designated period.

Characteristics	*N* = 1443^a^
MAP in the Aox‐period (mmHg)	39 (35 to 43)
MAP in the AoX‐period, *n* (%)	
> 40 mmHg	584 (40)
≤ 40 mmHg	859 (60)
MAP in the AoX‐period, *n* (%)	
> 50 mmHg	84 (5.8)
≤ 50 mmHg	1359 (94)
AoX duration (min)	49 (36 to 64)
Surgery duration^b^ (h)	4.28 (3.73 to 4.95)
Time in the AoX‐period with MAP < 40 mmHg (min)	28 (15 to 43)
Time in the AoX‐period with MAP < 50 mmHg (min)	44 (32 to 59)
Percent of AoX time with MAP < 40 mmHg (%)	64 (31 to 92)
Percent of AoX time with MAP < 50 mmHg (%)	100 (91 to 100)

*Note:* Definitions: AoX‐period: From aortic cross‐clamping to the removal of the aortic cross‐clamp. MAP in the AoX was defined as the average mean arterial pressure from the initiation of aortic cross‐clamping until removal of the aortic cross‐clamp; hypotension burden was quantified as the cumulative time and the proportion of total AoX duration spent below specified MAP thresholds (< 40 mmHg and < 50 mmHg).

Abbreviations: AoX, aortic cross‐clamp; CKMB, creatine kinase—myocardial band; MAP, mean arterial pressure; NE, norepinephrine.

^a^ Median (IQR); *n* (%).

^b^ Surgery duration: From induction to transfer to ICU.

Patients with an AoX duration above the median had higher postoperative CK‐MB levels than those with an AoX duration below the median (Figure 2). The postoperative CK‐MB level was 12% (95% CI: 10% to 14%; *p* < 0.001) higher per 15 min increase in AOX duration.

### 4.4 Hemodynamic Risk Factors Before and After AoX

#### 4.4.1 Before AoX

Patients treated with NE had a 16% higher postoperative CK‐MB level (95% CI: 6.0% to 26%; *p* < 0.01) than patients not treated with NE.

In the univariate analysis, the postoperative CK‐MB level was 6.0% (95% CI: −12% to −1.0%, *p* = 0.03) lower for each 10‐mmHg increase in MAP during the period before the AoX‐period. This result was not significant in the multivariable analysis (Table S3).

#### 4.4.2 After the AoX‐Period

After the AoX was removed, the relationship between MAP and the postoperative CK‐MB level reappeared. The postoperative CK‐MB concentration was 14% (95% CI: −20% to −8.0%, *p* < 0.001) lower for every 10 mmHg increase in MAP, and the postoperative CK‐MB concentration was 23% (95% CI: 15% to 31%; *p* < 0.001) higher when patients were treated with NE, compared to those not treated with NE (Table S4).

### 4.5 Other Surgical Risk Factors

The median surgery duration was 4.28 h (IQR: 3.73 to 4.95 h) (Table 2). For every 15 min of surgery duration, postoperative CK‐MB levels were 2.0% (95% CI: 0.0% to 3.0%; *p* = 0.03) higher, and undergoing valve surgery in addition to CABG resulted in a 52% (95% CI: 43% to 62%, *p* < 0.001) higher CK‐MB level postoperatively than patients only having CABG surgery. The change in CK‐MB level during the first 18 h postoperative was different between groups; CK‐MB level was 1.0% (95% CI: 0.0% to 1.0%; *p* < 0.001) higher for patients who had valve surgery in addition to CABG (Table S2), compared to patients who did not have valve surgery.

## 5 Discussion

We found a possible association between both surgical and hemodynamic parameters during open‐heart surgery with CPB and postoperative myocardial injury.

As expected, our results showed an increase in postoperative CK‐MB with longer AoX duration, indicating an association with myocardial injury.

Regarding hemodynamics, the results were complex: NE treatment during the AoX‐period was, in our analyses, associated with an increase in postoperative CK‐MB, indicating an association with myocardial injury, whereas MAP during the AoX‐period was not.

### 5.1 The Associations Between Myocardial Injury, NE, and MAP

Our results suggest that NE use during CPB may be associated with myocardial injury, an interesting finding given its status as a first‐choice vasopressor despite limited evidence [6]. Previously, NE has been associated with impaired organ perfusion and peripheral ischemia; however, it is important to notice that the relationship between the use of NE and organ perfusion is complex [11, 12, 13], and has been primarily studied in patients with septic shock [14]. A recent prospective study, regarding cardiac surgery patients with CPB, found that NE use was associated with a higher incidence rate of major postoperative events, including stroke, delirium, new‐onset atrial fibrillation or flutter, low cardiac output syndrome, acute kidney injury, sepsis, and in‐hospital death [15]. Additionally, NE has been associated with adverse cardiogenic events [8, 9]. This supports our findings; however, as our study is observational by nature, many factors may influence the association between NE and myocardial injury.

In contrast, no association was found between MAP during the AoX‐period and myocardial injury, suggesting MAP is less significant in this context. Previous studies comparing low and high MAP during CPB show conflicting results, leaving the optimal MAP target unclear [16, 17, 18, 19, 20, 21]. Across European heart centers, the recommended MAP target during open‐heart surgery varies [22]. In our study, the average MAP during the AoX period was 39 mmHg, which is lower compared to other centers and the European guidelines recommendations [23]. Although this may limit the external validity of our findings, it's worth noting that mortality was in line with reports from other centers [24, 25]. It is noteworthy that we found an association between MAP and postoperative CK‐MB in the periods before and after the AoX‐period. For every increase of 10 mmHg in MAP, postoperative CK‐MB decreased, which suggests a potential protective effect of a high MAP to avoid myocardial injury (Tables S3 and S4). In contrast, our interaction test between MAP and NE revealed that patients with high MAP who received NE had greater myocardial injury than patients with low MAP who did not receive NE. The interplay between NE and MAP underscores the complexity of hemodynamics during these operations and suggests that these variables may not be fully independent in their effects on myocardial injury. To draw definitive conclusions about their impact on myocardial injury, we require randomized controlled trials; one is currently ongoing and enrolling patients at Rigshospitalet (NCT05635227).

### 5.2 Risk of Myocardial Injury With Increasing AoX Duration

Prolonged AoX durations were associated with significant increases in postoperative CK‐MB levels, which indicate that time with AoX is a crucial factor regarding myocardial injury after heart surgery with CPB. Previous research has linked prolonged CPB duration with increased morbidity and mortality, particularly affecting pulmonary, renal, and neurological functions, though studies specifically on AoX duration are limited [26].

The significant increase in postoperative CK‐MB levels may be due to prolonged contact between blood and the mechanical surfaces of the bypass equipment. This has previously been associated with an inflammatory response that causes organ dysfunction. Furthermore, the AoX and subsequent ischemic reperfusion injury are known to be associated with significant activation of immune cells, including myocardial cytokines and white blood cells, leading to organ dysfunction [27, 28]. Patient comorbidities and surgical techniques could also contribute to elevated postoperative CK‐MB levels.

### 5.3 Strengths and Limitations

This study is strengthened by a standardized approach to patient care, a large sample size of all consecutive patients, high data quality, and complete follow‐up on the primary outcome. This center's post‐coronary artery surveillance for ischemia is based on CK‐MB measurements. Although high‐sensitive troponin T would have been more sensitive for detecting myocardial injury, CK‐MB was used in this study due to standard practice.

The observational design is inherently at risk of confounding and bias. However, by extracting data directly from electronic charts, we minimized interpersonal differences in data collection and eliminated recall bias due to the lack of direct patient contact. Furthermore, we acknowledge that NE administration was at the anesthesiologist's discretion, introducing a systematic bias such that patients receiving NE might have been more unstable. To minimize this bias, we adjusted our analyses for age, LVEF, and sex. Secondly, because our study is observational in nature, no causality can be inferred from our findings.

Consequently, our findings should be interpreted with caution, and we advocate for confirmatory randomized controlled trials to investigate the effects of different MAP targets and NE doses on postoperative CK‐MB levels to further explore and validate these results.

## 6 Conclusion

In this prospective observational study of open‐heart surgery with CPB, NE‐use during the AoX‐period seemed to predict myocardial injury, while MAP alone had no effect, highlighting the need for further studies.

### 6.1 Perspective

Future research should investigate the interplay of surgical and hemodynamic factors during CPB, particularly the use of NE during the AoX‐period as a predictor of myocardial injury. Randomized controlled trials are needed to clarify the individual and combined effects of these factors and to guide strategies for reducing myocardial injury.

## Author Contributions

**Julie T. Holm:** writing original draft, analysis, review and editing. **Sebastian C. Wiberg:** conceptualisation, review and editing. **Lars Grønlykke:** conceptualisation, review and editing. **Sarah E. Busch:** review and editing. **Peter Hasse Møller‐Sørensen:** conceptualisation, review and editing. **Christian Hassager:** conceptualisation, review and editing. **Theis S. Itenov:** conceptualisation, methodology, formal analysis, review and editing.

## Funding

The authors have nothing to report.

## Disclosure

To assist with language quality and spelling, the author used the automated spell‐ and grammar‐checking software Grammarly across the entire text.

## Ethics Statement

The study was assessed by the Research Ethics Committee of the Capital Region of Denmark (De Videnskabsetiske Komitéer i Region Hovedstaden), which determined that formal notification was not required for this observational study. Consequently, the need for informed consent was waived. IRB Ref No: F‐25010803.

## Consent

The authors have nothing to report.

## Conflicts of Interest

The authors declare no conflicts of interest.

## Supporting information


**Table S1:** Hemodynamics in the AoX‐period.
**Table S2:** Surgical risk factors for myocardial injury.
**Table S3:** Hemodynamics before the AoX‐period.
**Table S4:** Hemodynamics after the AoX‐period.
**Table S5:** Norepinephrine and mean arterial pressure interaction in the AoX‐period.
**Table S6:** Impact of norepinephrine dosage and mean arterial pressure on CK‐MB during the first 18 h postoperative.
**Figure S1:** Interaction between varying doses of norepinephrine and mean arterial pressure during the AoX period.

## Data Availability

Data available on request due to privacy/ethical restrictions.
