# Supplementary material for: Risk Factors for Myocardial Injury During Cardio‐Pulmonary Bypass‐Assisted Heart Surgery: A Retrospective Single‐Center Study
Source: Acta Anaesthesiol Scand. 2026 Sep 23;70(10):e70330. doi: 10.1111/aas.70330 (PMC13598622; doi:10.1111/aas.70330)
Supplement: Supplementary file 1 [file AAS-70-0-s001.docx]

# Supplementary

**Table S1:** **Hemodynamics in the AoX-period**

|  |  | **Univariate** | | | **Multivariable** | | |
| --- | --- | --- | --- | --- | --- | --- | --- |
|  | **Characteristics** | **Beta** | **95% CI^1^** | **p-value** | **Beta** | **95% CI^1^** | **p-value** |
| **MAP** | **Time from the end of surgery to CK-MB sample** | -0.02 | -0.03 to 0.01 | <0.001 | -0.02 | -0.03 to 0.01 | <0.001 |
|  | **MAP in the AoX-period, per 10 mmHg** | 0.03 | -0.02 to 0.09 | 0.20 | 0.03 | -0.02 to 0.09 | 0.20 |
|  | **Time from end of surgery to CK-MB sample and MAP in the AoX-period, per 10 mmHg** | 0.00 | 0.00 to 0.00 | 0.80 | 0.00 | 0.00 to 0.00 | 0.90 |
| **NE** | **Time from the end of surgery to CK-MB sample** | -0.02 | -0.02 to -0.02 | <0.001 | -0.02 | -0.02 to -0.02 | <0.001 |
|  | **NE in the AoX-period (ref: No)** |  |  |  |  |  |  |
|  | Yes | 0.19 | 0.09 to 0.29 | <0.001 | 0.17 | 0.07 to 0.27 | <0.001 |
|  | **Time from the end of surgery to CK-MB sample and NE in the AoX-period** | 0.00 | -0.01 to 0.00 | 0.70 | 0.00 | -0.01 to 0.00 | 0.70 |
|  |  |  |  |  |  |  |  |

^1^ CI = Confidence Interval

**Abbreviations**: MAP: Mean arterial pressure; NE: Norepinephrine; CKMB: Creatine Kinase – myocardial band; AoX: Aortic Cross-Clamp.

**Model structure**: Data are presented as estimates from univariate linear mixed-effects models. CK-MB values were log-transformed before analysis; thus, estimates represent the relative change in CK-MB levels. The models include interaction terms between time (hours post-surgery) and each surgical characteristic to assess their impact on the kinetic profile of CK-MB. MAP in the AoX-period is analyzed in 10 mmHg increments. The multivariable analysis was adjusted for age, sex, and pre-operative left ventricular ejection fraction.

**Variables:** MAP median (cut-off) = 39 mmHg; AoX duration median (cut-off) 49 minutes

**Definitions:** Time from the end of surgery to CK-MB sample: The first 18 hours postoperative; AoX-period: From aortic cross-clamping to the removal of the aortic cross-clamp

## **Table S2: Surgical risk factors for myocardial injury**

|  |  | Univariate | | | Multivariable | | |
| --- | --- | --- | --- | --- | --- | --- | --- |
|  | Characteristics | Beta | **95% CI^1^** | **p-value** | Beta | **95% CI^1^** | **p-value** |
| **AoX duration** | **Time from the end of surgery to CK-MB sample** | -0.03 | -0.03 to -0.02 | <0.001 | -0.03 | -0.03 to -0.02 | <0.001 |
|  | **AoX duration, per 15 minutes** | 0.12 | 0.10 to 0.14 | <0.001 | 0.12 | 0.10 to 0.13 | <0.001 |
|  | **Time from the end of surgery to CK-MB sample * Time with AoX, per 15 minutes** | 0.00 | 0.00 to 0.00 | <0.01 | 0.00 | 0.00 to 0.00 | <0.01 |
| **CABG/Valve** | **Time from the end of surgery to CK-MB sample** | -0.02 | -0.02 to -0.02 | <0.001 | -0.02 | -0.02 to -0.02 | <0.001 |
|  | **CABG and valve surgery (ref: No)** |  |  |  |  |  |  |
|  | **Yes** | 0.52 | 0.43 to 0.62 | <0.001 | 0.50 | 0.41 to 0.60 | <0.001 |
|  | **Time from end of surgery to CK-MB sample * CABG and valve surgery (Yes)** | 0.01 | 0.00 to 0.01 | <0.001 | 0.01 | 0.00 to 0.01 | <0.001 |
| **Surgery time** | **Time from the end of surgery to CK-MB sample** | -0.02 | -0.03 to -0.02 | <0.001 | -0.02 | -0.03 to -0.02 | <0.001 |
|  | **Surgery duration, per 15 minutes** | 0.02 | 0.00 to 0.003 | 0.03 | 0.02 | 0.00 to 0.003 | 0.03 |
|  | **Time from the end of surgery to CK-MB sample * Surgery duration, per 15 minutes** | 0.00 | 0.00 to 0.00 | 0.30 | 0.00 | 0.00 to 0.00 | 0.30 |

^1^ CI = Confidence Interval

**Abbreviations**: AoX: aortic cross-clamp; CI: confidence interval; CK-MB: creatine kinase myocardial band; MAP: mean arterial pressure; NE: norepinephrine;

**Model structure:** Data are presented as estimates from univariate and multivariable linear mixed-effects models. CK-MB values were log-transformed before analysis; thus, estimates represent the relative change in CK-MB levels. The models include interaction terms between time (Time from the end of surgery to CK-MB sample) and each surgical characteristic to assess their impact on the kinetic profile of CK-MB. AoX duration and surgery duration are analyzed in 15-minute increments. Combined surgery (CABG + valve) is compared against isolated procedures.The multivariable analysis was adjusted for age, sex, and pre-operative left ventricular ejection fraction.

**Variables**: AoX duration median (cut-off) 49 minutes; Surgery duration median (cut-off) 4.28 hours

**Definitions:** Time from end of surgery to CK-MB sample: The first 18 hours postoperative; Time with AoX: From aortic cross-clamping to the removal of the aortic cross-clamp; Surgery duration: From induction to transfer to ICU

**Table S3: Hemodynamics before the AoX-period**

|  |  | **Univariate** | | | **Multivariable** | | |
| --- | --- | --- | --- | --- | --- | --- | --- |
|  | **Characteristics** | **Beta** | **95% CI^1^** | **p-value** | **Beta** | **95% CI^1^** | **p-value** |
| **MAP** | **Time from the end of surgery to CK-MB sample** | -0.02 | -0.03 to 0.00 | <0.001 | -0.02 | -0.03 to 0.00 | 0.01 |
|  | **MAP before the AoX-period, per 10 mmHg** | -0.06 | -0.12 to -0.01 | 0.02 | -0.05 | -0.10 to 0.01 | 0.09 |
|  | **Time from the end of surgery to CK-MB sample and MAP before the AoX-period, per 10 mmHg** | 0.00 | 0.00 to 0.00 | 0.80 | 0.00 | 0.00 to 0.00 | 0.80 |
| **NE** | **Time from the end of surgery to CK-MB sample** | -0.02 | -0.02 to -0.02 | <0.001 | -0.02 | -0.02 to -0.02 | <0.001 |
|  | **NE before the AoX-period (ref: No)** |  |  |  |  |  |  |
|  | Yes | 0.16 | 0.06 to 0.26 | <0.01 | 0.14 | 0.04 to 0.24 | <0.01 |
|  | **Time from the end of surgery to CK-MB sample and NE before the AoX-period (Yes)** | 0.00 | -0.01 to 0.00 | 0.03 | 0.00 | -0.01 to 0.00 | 0.03 |

^1^ CI = Confidence Interval

**Abbreviations**: MAP: Mean arterial pressure; NE: Norepinephrine; CKMB: Creatine Kinase – myocardial band; AoX: Aortic Cross-Clamp.

**Model structure**: Data are presented as estimates from univariate and multivariable linear mixed-effects models. CK-MB values were log-transformed before analysis; thus, estimates represent the relative change in CK-MB levels. The models include interaction terms between time (time from the end of surgery) and each hemodynamic characteristic to assess their impact on the kinetic profile of CK-MB. MAP before the AoX-period is analyzed in 10 mmHg increments. The multivariable analysis was adjusted for age, sex, and pre-operative left ventricular ejection fraction.

**Definitions:** Time from the end of surgery to CK-MB sample: The first 18 hours postoperative; AoX-period: From aortic cross-clamping to the removal of the aortic cross-clamp

## **Table S4: Hemodynamics after the AoX-period**

|  |  | **Univariate** | | | **Multivariable** | | |
| --- | --- | --- | --- | --- | --- | --- | --- |
|  | **Characteristics** | **Beta** | **95% CI^1^** | **p-value** | **Beta** | **95% CI^1^** | **p-value** |
| **MAP** | **Time from the end of surgery to CK-MB sample** | -0.02 | -0.03 to 0.00 | 0.02 | -0.02 | -0.03 to 0.00 | 0.02 |
|  | **MAP after the AoX-period, per 10 mmHg** | -0.14 | -0.20 to 0.01 | <0.001 | -0.12 | -0.18 to -0.06 | <0.001 |
|  | **Time from end of surgery to CK-MB sample and MAP after the AoX-period, per 10 mmHg** | 0.00 | 0.00 to 0.00 | 0.60 | 0.00 | 0.00 to 0.00 | 0.70 |
| **NE** | **Time from the end of surgery to CK-MB sample** | -0.02 | -0.02 to -0.02 | <0.001 | -0.02 | -0.02 to -0.02 | <0.001 |
|  | **NE after the AoX-period (ref: No)** |  |  |  |  |  |  |
|  | Yes | 0.23 | 0.15 to 0.31 | <0.001 | 0.21 | 0.13 to 0.29 | <0.001 |
|  | **Time from the end of surgery to CK-MB sample and NE after the AoX-period (Yes)** | 0.00 | 0.00 to 0.00 | 0.40 | 0.00 | 0.00 to 0.00 | 0.40 |

^1^ CI = Confidence Interval

**Abbreviations**: MAP: Mean arterial pressure; NE: Norepinephrine; CKMB: Creatine Kinase – myocardial band; AoX: Aortic Cross-Clamp.

**Model structure**: Data are presented as estimates from univariate and multivariable linear mixed-effects models. CK-MB values were log-transformed before analysis; thus, estimates represent the relative change in CK-MB levels. The models include interaction terms between time (Time from the end of surgery to CK-MB sample) and each hemodynamic characteristic to assess their impact on the kinetic profile of CK-MB. MAP after the AoX-period is analyzed in 10 mmHg increments. The multivariable analysis was adjusted for age, sex, and pre-operative left ventricular ejection fraction.

**Definitions:** Time from the end of surgery to CK-MB sample: The first 18 hours postoperative; AoX-period: From aortic cross-clamping to the removal of the aortic cross-clamp

**Table S5: Norepinephrine and mean arterial pressure interaction in the AoX-period**

|  | **Characteristics** | **Beta** | **95% CI^1^** | **p-value** |
| --- | --- | --- | --- | --- |
| **Main effects** | **Time from the end of surgery to CK-MB sample** | -0.02 | -0.02 to -0.02 | <0.001 |
| **NE** | **NE in the AoX-period (ref: No)** |  |  |  |
|  | Yes | 0.17 | 0.03 to 0.32 | 0.016 |
| **MAP** | **MAP in the AoX-period (ref: MAP below median)** |  |  |  |
|  | Above median | 0.04 | -0.04 to 0.12 | 0.34 |
| **Two-way interactions** |  |  |  |  |
| **Time and NE** | **Time from the end of surgery to CK-MB sample * NE in the AoX-period** | 0.00 | -0.01 to 0.01 | 0.89 |
| **Time and MAP** | **Time from the end of surgery to CK-MB sample * MAP (above median) in the AoX-period** | 0.00 | -0.01 to 0.00 | 0.33 |
| **NE and MAP** | **NE in the AoX-period * MAP (above median) in the AoX-period** | 0.02 | -0.18 to 0.22 | 0.82 |
| **Three-way interaction** |  |  |  |  |
| **Time, NE, and MAP** | **Time from the end of surgery to CK-MB sample** * **NE in the AoX-period * MAP (above median) in the AoX-period** | 0.00 | -0.01 to 0.01 | 0.67 |
|  |  |  |  |  |
|  |  |  |  |  |

^1^ CI = Confidence Interval

**Abbreviations:** MAP: Mean arterial pressure; NE: Norepinephrine; CK-MB: Creatine Kinase – myocardial band; AoX: Aortic Cross-Clamp.

**Model structure**: Data are presented as estimates from univariate linear mixed-effects models. CK-MB values were log-transformed before analysis; thus, estimates represent the relative change in CK-MB levels. The models include interaction terms between time (Time from the end of surgery to CK-MB sample) and each hemodynamic characteristic (NE in the AoX-period: yes/no; MAP in the AoX-period: above/below median) to assess their impact on the kinetic profile of CK-MB.

**Definitions**: Time from the end of surgery to CK-MB sample: The first 18 hours postoperative; AoX-period: From aortic cross-clamping to the removal of the aortic cross-clamp.

**Table S6: Impact of norepinephrine dosage and mean arterial pressure on CK-MB during the first 18 hours postoperative.**

|  | **Characteristics** | **Beta** | **95% CI^1^** | **p-value** |
| --- | --- | --- | --- | --- |
|  | **Time from the end of surgery to CK-MB sample** | -0.02 | -0.02, -0.02 | <0.001 |
| **NE** | **NE in the AoX-period (**µg/kg/min) |  |  | <0.001 |
|  | [0 to 0.01] | — | — |  |
|  | [0.01 to 1] | 0.18 | 0.08, 0.29 |  |
|  | [≥1] | 0.23 | 0.08, 0.38 |  |
| **MAP** | **MAP in the AoX-period (ref: MAP below median)** |  |  | 0.6 |
|  | Above median | 0.03 | -0.07, 0.12 |  |
| **Two-way interactions** |  |  |  |  |
| **Time and NE** | **Time from the end of surgery to CK-MB sample * NE in the AoX-period** [µg/kg/min] (**ref:** [0,0.01]) |  |  | 0.034 |
|  | **Time from the end of surgery to CK-MB sample** * [0.01,1] | 0.01 | 0.00, 0.01 |  |
|  | **Time from the end of surgery to CK-MB sample** * [≥1] | 0.00 | -0.01, 0.00 |  |
| **Time and MAP** | **Time from the end of surgery to CK-MB sample * MAP (above median) in the AoX-period** | 0.00 | 0.00, 0.01 | 0.4 |
| **NE and MAP** | **NE in the AoX-period * MAP (above median) in the AoX-period (ref: MAP (above median) *** [0,0.01]) |  |  | 0.8 |
|  | **MAP (above median) *** [0.01,1] | 0.01 | -0.15, 0.18 |  |
|  | **MAP (above median) *** [≥1] | 0.00 | -0.14, 0.32 |  |
| **Three-way interaction** |  |  |  |  |
| **Time, NE, and MAP** | **Time from the end of surgery to CK-MB sample** * **NE in the AoX-period * MAP (above median) in the AoX-period** |  |  | 0.069 |
|  | **Time from the end of surgery to CK-MB sample * MAP (above median) *** [0.01,1] | -0.01 | -0.02, 0.00 |  |
|  | **Time from the end of surgery to CK-MB sample * MAP (above median) *** [≥1] | 0.00 | -0.01, 0.01 |  |

^1^ CI = Confidence Interval

**Abbreviations:** MAP: Mean arterial pressure; NE: Norepinephrine; CK-MB: Creatine Kinase – myocardial band; AoX: Aortic Cross-Clamp.

**Model structure:** Data are presented as estimates from univariate linear mixed-effects models. CK-MB values were log-transformed before analysis; thus, estimates represent the relative change in CK-MB levels. The models include interaction terms between time (Time from the end of surgery to CK-MB sample), MAP in the AoX-period (above/below median), and NE dosage in the AoX-period (categorized as: [0 to 0.01], [0.01 to 1], and [>1] µg/kg/min) to assess their impact on the kinetic profile of CK-MB. The lowest NE dose interval [0 to 0.01 µg/kg/min] and MAP below the median serve as reference categories.

**Definitions**: Time from the end of surgery to CK-MB sample: The first 18 hours postoperative; AoX-period: From aortic cross-clamping to the removal of the aortic cross-clamp.

## **Figure S1: Interaction between varying doses of norepinephrine and mean arterial pressure during the AoX period**

**Abbreviations:** MAP: Mean arterial pressure; NE: Norepinephrine; CK-MB: Creatine Kinase – myocardial band; AoX: Aortic Cross-Clamp.

**Model structure**: MAP was treated as a categorical variable, below and above the median, and NE doses were separated into three dose intervals: [0 to 0.01], [0.01 to 1], and [>1] µg/kg/min. CK-MB values were log-transformed before analysis; thus, estimates represent the relative change in CK-MB levels.

**Definition:** Time to sample (hours): Time from the end of surgery to CK-MB sample, which is the first 18 hours postoperative.
